# Histopathological review of prostate cancer in Lokoja, Nigeria

**DOI:** 10.1186/1750-9378-6-S1-A2

**Published:** 2011-08-11

**Authors:** Olabode Peter Oluwole, Jones Olaoluwa Taiwo, Kinsley U Awani, Emmanuelle O Adugba

**Affiliations:** 1 Department of Pathology, College of Health Sciences, University of Abuja, Nigeria; 2 Department of Surgery, Federal Medical Centre, Lokoja, Nigeria; 3 Departmernt of Surgery. Kogi Specialist Hospital, Lokoja, Nigeria

## Background

Prostate cancer is a major health problem among middle aged and elderly men, though its incidence and mortality varies Worldwide. Although, earlier reports within Africa Continent suggested prostate cancer to be rare among African men, it is now reported to be a surprisingly common disease.

## Objective

The objective of this review is to characterize and determine the pattern of distribution of prostate cancer in our environment.

## Materials and methods

This is a 2-year retrospective histopathological review of prostate cancer diagnosed during August 2007 to July 2009, in the Department of Histopathology of Federal Medical Centre, Lokoja. After histological assessment, the tumours were classified according to WHO recommendation. Histological grading and scoring of adenocarcinoma was done using Gleason system.

## Results

Eight (8) cases of prostate cancer were analyzed. The age range was between 45-80 years, with a mean of 60 years. All the surgical biopsies were trucut needle biopsy. Six (75%) cases were poorly differentiated and two (25%) were moderately differentiated adenocarcinomas, while neither well differentiated adenocarcinoma nor other histological types was seen.

## Conclusion

Though the number of patients in this review was small, it showed that most of the patients presented with advanced disease. The factors may be that the histopathology services is new in the Hospital and its environs, lack of health education, absence of routine screening programmes including PSA estimation, inadequate diagnostic facilities and abject poverty.

## Acknowledgements

Publication of this article was funded in part by the University of Florida Open-Access Publishing Fund.

